# P-1387. Barriers to PrEP Implementation in Publicly Funded Family Planning Clinics in Atlanta, Georgia

**DOI:** 10.1093/ofid/ofae631.1563

**Published:** 2025-01-29

**Authors:** Lily Bonadonna, Katherine M Anderson, Deja Er, Jessica Sales, Anandi N Sheth

**Affiliations:** Emory University School of Medicine Department of Internal Medicine, ATLANTA, Georgia; Emory University Rollins School of Public Health, Atlanta, Georgia; Emory University School of Medicine, Atlanta, Georgia; Emory University, Rollins School of Public Health, Atlanta, Georgia; Emory University School of Medicine, Atlanta, Georgia

## Abstract

**Background:**

Although Georgia has the highest HIV incidence in the US, uptake of pre-exposure prophylaxis (PrEP) for HIV is inadequate, especially among cisgender (cis) women. The Title X Family Planning Program (FP) provides federal funding for sexual health services to low income or uninsured individuals. We sought to understand clinic interest in and capacity for women-centered PrEP care implementation in Title X FP clinics in metro Atlanta.

**Figure:**
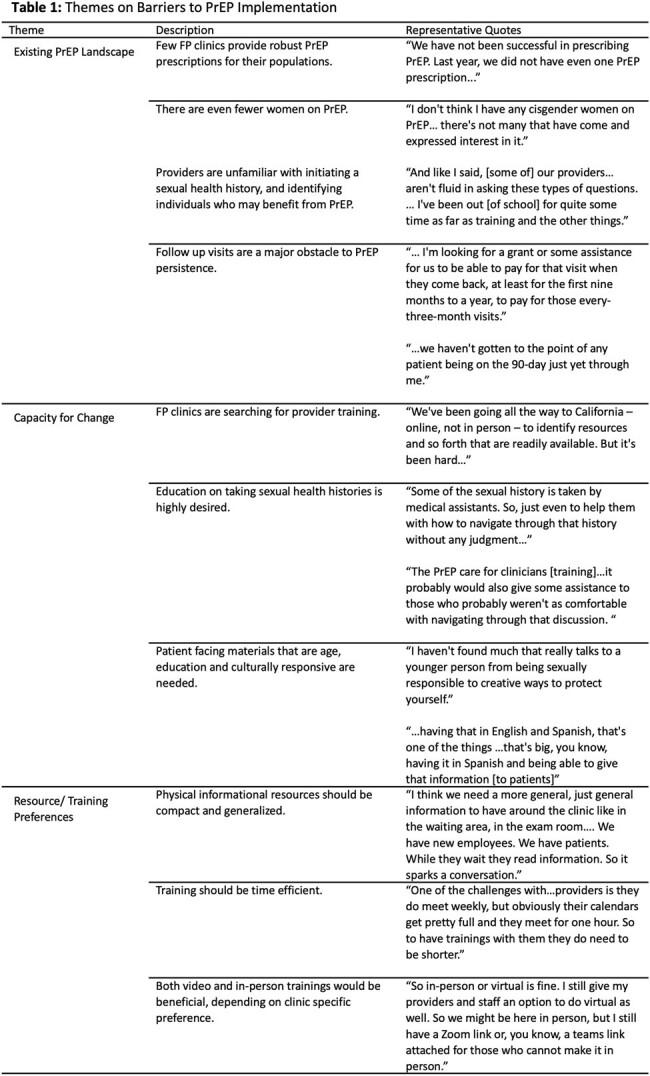

**Methods:**

From Jan 2022-Nov 2023, we held focus groups with 47 participants representing 22 FP clinics across four metro Atlanta *Ending the HIV Epidemic* priority counties (71% of all Title X FP clinics in the area). We used a structured interview guide to assess PrEP implementation strategies across each step of PrEP care; participants included advanced practice providers, medical assistants, nurses, and FP program leaders. Following standard rapid qualitative analytic methods, two trained researchers created a matrix of responses by question, assessed salience of responses by deductive themes, and used memos to summarize themes for narrative description.

**Results:**

Clinics had few-to-no cis women on PrEP. Conversations regarding PrEP eligibility were often patient-initiated, rather than provider-initiated. Many providers expressed unfamiliarity with sexual history taking and assessing HIV risk, especially with cis women. Providers also cited patient barriers to PrEP uptake and persistence, including insurance navigation and follow-up visit costs. All providers expressed a need for time-efficient screening and counseling procedures and trainings covering sexual history taking, destigmatizing PrEP, and medication access. Population-tailored information, such as non-English, youth-focused, and low literacy responsive materials were highly desired (Table 1).

**Conclusion:**

Family planning clinics in Atlanta require comprehensive training across the steps of PrEP care, given limited provider initiation of and comfort with risk assessment and counseling, and perceived barriers to uptake and retention. Multi-pronged implementation strategies are necessary to prevent new HIV infections, particularly among cis women, and to progress toward ending the HIV epidemic.

**Disclosures:**

**All Authors**: No reported disclosures

